# Quality of Life in Adolescents with Idiopathic Scoliosis: A Longitudinal Study Exploring the Impact of Psychological Distress, Joint Hypermobility, Body Mass Index, and Physical Activity

**DOI:** 10.3390/jcm15145422

**Published:** 2026-07-10

**Authors:** Dalia Alimam, Nour Alhafdhi, Lolwah Alrashed Alhumaid, Samia A. Alamrani, Abdulaziz Bin Shebreen

**Affiliations:** 1 Department of Rehabilitation Sciences, College of Applied Medical Sciences, King Saud University, Riyadh 10219, Saudi Arabia; lalrashed@ksu.edu.sa; 2 Department of Physical Therapy, King Fahad Medical City, Riyadh Second Health Cluster, Riyadh 12231, Saudi Arabia; nalhafdhi@gmail.com; 3 Department of Health Rehabilitation Sciences, Faculty of Applied Medical Sciences, University of Tabuk, Tabuk 71491, Saudi Arabia; salamrani@ut.edu.sa; 4 Spine Surgery Department, King Fahad Medical City, Riyadh Second Health Cluster, Riyadh 12231, Saudi Arabia; dr.aziz_2006@hotmail.com; 5 National Neurosciences Institute, Riyadh 11525, Saudi Arabia

**Keywords:** idiopathic scoliosis, quality of life, psychological distress, physical activity, body mass index

## Abstract

**Objective:** The objective of this study was to examine the association between baseline psychological distress, joint hypermobility, body mass index (BMI), and physical activity with Quality of Life (QoL) in adolescents with Idiopathic Scoliosis (AIS) 12 months on. **Design:** This is a prospective longitudinal study. Adolescents (10–18 years) with AIS were recruited. At baseline (n = 69), participants completed questionnaires covering demographics, psychological distress (Depression, Anxiety and Stress Scale, DASS-21), physical activity (International Physical Activity Questionnaire, IPAQ), and QoL (SRS-22r). Clinical assessments included joint hypermobility and BMI measurement. After 12 months, participants (n = 49) completed the SRS-22r to re-evaluate QoL. Factors associated with QoL were explored using bivariate and multiple linear regression analyses. **Results:** Multiple linear regression models assessing factors associated with QoL 12 months after baseline measurements explained 53.3% of variance in SRS-22 scores (*p* < 0.001), with an adjusted R^2^ of 44.6%. Among baseline explanatory variables, thoracolumbar/lumbar Cobb angle contributed most significantly (β = 0.476, *p* < 0.05), followed by BMI (β = 0.342, *p* < 0.05) and DASS-Stress scores (β = −0.309, *p* < 0.05). **Conclusions:** Cobb angle, BMI, and stress emerged as important factors associated with QoL, highlighting the importance of addressing both clinical and psychosocial factors in AIS care.

## 1. Introduction

Adolescent idiopathic scoliosis (AIS), the most common type of spinal deformity in adolescents, is characterized by a lateral curvature of the spine that often develops during youth [1]. AIS affects approximately 1–4% of adolescents, with girls at a notably higher risk of curve progression [1]. In Riyadh, the capital of Saudi Arabia, a 2022 school-based study (based solely on scoliometer readings, without confirmatory X-rays) of 591 adolescents aged 12–18 indicated that AIS might occur in 29.4% of participants [2].

Although AIS is primarily characterized by spinal deformity, growing evidence from the broader musculoskeletal pain literature (e.g., low back pain) indicates that psychosocial factors substantially influence pain, disability, and health-related outcomes [3]. Accordingly, a biopsychosocial approach has been increasingly advocated to better understand the determinants of patient-reported outcomes in AIS beyond radiographic measures alone [4]. Consequently, contemporary AIS management aims to address not only structural deformity but also its physical, psychological, and social impacts. In particular, consideration of psychosocial aspects such as health-related quality of life (QoL) is necessary because these can be significantly affected by the deformity and its treatment [5]. A complex interplay exists between physical and psychological factors on the QoL of adolescents with AIS. Psychological distress, such as stress and depression [6], has been linked to poorer function and QoL outcomes, identifying the need to address mental health alongside physical management in AIS management. Additionally, physical factors such as joint hypermobility, body mass index (BMI), and habitual physical activity levels may also influence QoL, directly or through their impact on physical health and self-perception [5,7].

How physical and psychological factors interact over time in the treatment of AIS has not been intensively investigated. Associations between QoL and psychological distress, joint hypermobility, BMI, and physical activity levels in patients 12 months after an initial baseline survey are herein explored. These associations improve understanding of AIS management, and allow tailored psychosocial and physical assessments to inform personalized interventions to improve long-term QoL.

### Study Aim

To investigate whether baseline psychological distress, joint hypermobility, BMI, and habitual physical activity levels are associated with QoL (12 months after baseline measurements) in patients with AIS.

## 2. Materials and Methods

### 2.1. Setting and Participants

A prospective cohort study was performed between 8 December 2023 and 11 March 2025. Adolescents diagnosed with AIS—defined as lateral curvature of the spine > 10° with a rotational component of the vertebrae [8]—were enrolled from King Fahd Medical City (KFMC) in Riyadh, Saudi Arabia. Study participation eligibility criteria included a confirmed clinical diagnosis of AIS with a Cobb angle ≥ 10°, age between 10 and 18 years, and signed informed electronic consent from both the adolescent and their legal guardian. Participants were then screened by a researcher (N.A.) using a standardized form to exclude individuals who had other scoliosis types (e.g., congenital or neuromuscular) or a history of recent spinal injuries or surgeries. The final diagnosis was confirmed by a consultant spine deformity surgeon. In accordance with Strengthening the Reporting of Observational Studies in Epidemiology (STROBE) guidelines, the study methodology, findings, and implications are detailed to enhance the clarity and reproducibility of observational research [9].

### 2.2. Measurements

Explanatory variables at baseline:

1. Demographic and clinical characteristics: Basic demographic information, including age and sex, was recorded using a standardized data-collection form. Clinical variables included whether the participant used a brace (e.g., thoracolumbosacral orthosis (TLSO)) for AIS (yes/no), attendance at follow-up physiotherapy sessions, use of any related medications, and the presence of comorbidities such as diabetes mellitus, cardiovascular disease, respiratory illnesses (e.g., asthma), and/or back pain. Back pain severity in the last week was measured using the Numeric Rating Scale (NRS), with participants rating their pain from 0 (none) to 10 (worst imaginable) [10].
2. Physical factors: Joint hypermobility was assessed using the Beighton scoring system during the clinical visit. This evaluation, conducted by N.A., involved nine physical maneuvers designed to test flexibility in specific joints. Each positive maneuver was scored, with a maximum total score of 9. A score of 0–3 indicates normal flexibility, while that ≥ 4 is considered to indicate generalized joint hypermobility [11,12]. BMI was also measured and calculated using the standard formula: weight divided by height squared (kg m^−2^). For the BMI classification, Centers for Disease Control and Prevention standards served as reference values [13]. BMI-for-age is classified as underweight (<5th percentile), healthy weight (5th–85th percentile), overweight (85th–95th percentile), obese (≥95th percentile) and severe obesity (120% of the 95th percentile or greater, or 35 kg/m^2^ or greater).
3. To assess habitual physical activity levels, the short-form Arabic version of the International Physical Activity Questionnaire (IPAQ) was used [14]. This tool records the frequency and duration of vigorous activity, moderate activity, walking, and sitting over the past seven days, measured in metabolic equivalent (MET) min per week. Scoring followed IPAQ guidelines; participants were categorized into low (not meeting minimum criteria), moderate (≥600 MET-min/week), and high (≥3000 MET-min/week) levels [14]. Cobb angle data were obtained from patients’ medical records. Severity was interpreted following 2016 SOSORT guidelines: a 10–20° Cobb angle was considered mild, 21–40° moderate, and >40° severe [15].
4. Psychological factors: Emotional well-being was evaluated using the Arabic version of the Depression, Anxiety, and Stress Scale (DASS-21), a validated self-report measure [16]. Participants rated the frequency of specific symptoms experienced over the previous 7 days on a 4-point Likert scale (0 = did not apply to me at all, to 3 = applied to me very much or most of the time). Severity classifications for each domain included: depression (normal 0–9, mild 10–13, moderate 14–20, severe 21–27, and extremely severe ≥ 28), anxiety (normal 0–7, mild 8–9, moderate 10–14, severe 15–19, and extremely severe ≥ 20), and stress (normal 0–14, mild 15–18, moderate 19–25, severe 26–33, and extremely severe ≥ 34). Higher scores across all subscales reflect greater levels of psychological distress.

Outcome variables 12 months after baseline measurement.

The primary outcome measure for assessing QoL in adolescents with AIS was the Arabic version of the Scoliosis Research Society-22r questionnaire [17]. This validated tool evaluates the key domains of pain, function, mental health, and self-image. Each item is rated on a 5-point Likert scale, where 1 indicates the poorest outcome and 5 the most favorable. Domain scores represent the average of responses within each respective domain; an overall QoL score is obtained by averaging all item responses; higher scores indicate a better QoL. Strong psychometric properties of the Arabic SRS-22r (good internal consistency, excellent test–retest reliability (ICC range 0.82–0.90), and acceptable concurrent validity) make it a reliable and culturally appropriate measure for Arabic-speaking adolescents with AIS.

### 2.3. Procedure

At baseline (n = 69), participants provided demographic and clinical data and completed the self-reported questionnaires [18]. The follow-up questionnaire was administered either in person or through a virtual clinic, based on participant preference. Follow-up assessments were conducted 12 months after baseline, with only minor scheduling variations of a few days around the target date. Participants were then asked to complete detailed questionnaires with the mandatory presence of N.A. to encourage answers, provide any necessary clarification, and to prevent a participant’s family from influencing any answer. Non-responders were followed up with a maximum of five contact attempts via text message or phone.

The study was conducted according to the guidelines of the Declaration of Helsinki. Ethical approval for the study was obtained from the Institutional Review Board at KFMC (IRB 22545, approval date 27 November 2022). All study procedures were conducted in accordance with the Declaration of Helsinki as developed by the World Medical Association.

### 2.4. Determining Sample Size

This study involved an initial cross-sectional phase [18] and a subsequent longitudinal phase (herein). The cross-sectional phase explored baseline characteristics of adolescents with idiopathic scoliosis. This longitudinal phase focuses on examining associations between baseline psychological and physical factors and follow-up (12 months on) QoL outcomes.

For the cross-sectional phase, sample size was calculated using the Raosoft online calculator, based on an estimated population of approximately 80 AIS patients treated at KFMC. Using a 50% response distribution, 95% confidence level, and 5% margin of error, the required sample size was determined to be 69 participants. All 69 participants were successfully enrolled. No separate sample size calculation was performed for the longitudinal phase.

### 2.5. Statistical Analysis

Data analysis was performed using IBM SPSS Statistics for Windows, v30.0 (IBM Corp., Armonk, NY, USA). Descriptive statistics summarized participant demographic and clinical characteristics. Continuous variables are presented as means and standard deviations (SD) or medians and interquartile ranges (IQR), depending on data distribution. Categorical variables are expressed as frequencies and percentages. The normality of continuous data was evaluated by Shapiro–Wilk tests, complemented by visual inspection of histograms and Q–Q plots. Baseline participant characteristics (demographics, clinical, physical, and psychological features) were compared between respondents and nonrespondents using independent samples *t*-tests or Mann–Whitney *U* tests for continuous variables, and χ^2^-tests or Fisher’s exact tests for categorical variables.

As an exploratory analysis, unadjusted correlations between the explanatory variables and the outcome were examined using Spearman’s rank correlation coefficients for continuous variables and point-biserial correlations for dichotomous variables. Bivariate linear regression analyses were then performed as the primary unadjusted analysis to examine the associations between each explanatory variable and the outcome (QoL).

The same explanatory variables, selected on the basis of clinical relevance and previous literature, were entered simultaneously into the initial multiple linear regression model, with age included as a control variable. Backward elimination was then used to derive a more parsimonious model. A sensitivity analysis was performed to evaluate the robustness of the final model. The regression model was rerun after excluding the non-significant explanatory variables, and the unstandardized coefficients, R^2^, and adjusted R^2^ values were compared between the full and reduced models [19]. Assumptions of the final model were assessed for homoscedasticity, normality, and independence of residuals. Multicollinearity was evaluated using variance inflation factor (VIF) values and correlation coefficients among explanatory variables. VIF values > 10 and correlation coefficients ≥ 0.70 were considered indicative of problematic multicollinearity. The regression analyses were exploratory in nature and intended to identify potential associations rather than establish causal relationships. For all statistical analyses, a two-tailed *p* value < 0.05 was considered statistically significant.

## 3. Results

Of the 69 baseline participants, 49 (71%) completed the 12-month follow-up assessment. Baseline characteristics of respondents and non-respondents are presented in Table 1. No significant differences existed between them for any baseline variable (*p* ≥ 0.05).

The median age of follow-up participants was 15 years (IQR 3); 83.7% were female. They reported moderate low back pain (LBP) intensity, with a median NRS score of 5 (IQR 5); 14.3% reported analgesic use. No participant smoked, and comorbidities were rare: one reported diabetes and another a respiratory condition. Psychological assessment indicated normal levels of stress and depression, with mild levels of anxiety (Table 1).

The median Beighton score of 6 (IQR 4) suggests generalized joint hypermobility (Table 1). While 49% of participants fell within a healthy weight BMI range, 30.6% were underweight. Physical activity levels were generally low (67.3% of participants); only 16.3% of participants engaged in regular weekly activity. Physiotherapy use was limited; 22.4% of participants reported having current PT sessions, mostly general exercise programs (18.4%).

Participants had a median thoracic angle of 40° (n = 28, IQR = 19.5) and a median thoracolumbar/lumbar Cobb angle of 31° (n = 33, IQR = 21.5), suggesting moderate scoliosis severity. Some participants contributed to both curve categories. Values for non-respondents were 48° (n = 9, IQR = 49.5) and 30.5° (n = 16, IQR = 22.3), respectively; there were no significant differences between respondents and non-respondents for either (*p* = 0.901 and 0.932, respectively).

Participant QoL 12 months after the baseline survey, assessed using the SRS-22 and its subscales, revealed scoliosis to have moderately affected it, with median (+ IQR) scores for each 5-point subscale being: function (3.7 (0.9)), pain (3.8 (0.8)), self-image (3.8 (1.4)), mental health (3.8 (1.4)), and satisfaction (4 (1.5)).

Correlation analysis (Appendix A, Table A1) revealed a significant, positive, moderate correlation between QoL after 12 months and thoracolumbar/lumbar Cobb angle (*r* = 0.361; *p* ≤ 0.05). Additionally, bivariate linear regression analyses (Table 2) indicated both the baseline thoracolumbar/lumbar Cobb angle and BMI to independently and significantly associated with 12-month QoL scores (*p* ≤ 0.05), accounting for 20.1% and 9.5% of the total variance in QoL, respectively. Regression analyses were the primary analyses used to examine associations with 12-month QoL. Accordingly, although BMI was not significantly correlated with QoL, it showed a significant association in the bivariate regression analysis. No significant associations existed between QoL and other baseline sociodemographic, clinical, psychological, or physical outcomes (*p* ≥ 0.05).

Thoracolumbar/lumbar Cobb angle data at follow-up were available for 33 of the 49 participants, and analyses were therefore based on this subset.

The initial multiple linear regression model examining baseline explanatory variables associated with QoL 12 months after the baseline survey explained 53.3% of the variance in SRS-22 scores (*p* < 0.001; Table 3); indicating that the model accounted for more than half of the variability in QoL. Among the baseline explanatory variables, the thoracolumbar/lumbar Cobb angle showed the strongest association with QoL (β = 0.476, *p* < 0.05), followed by BMI (β = 0.342, *p* < 0.05), and DASS-Stress scores (β = −0.309, *p* < 0.05). Age (a control variable) did not associate significantly with QoL (*p* ˃ 0.05). Although LBP severity (measured using NRS) was associated negatively with QoL (β = −0.235), this association was not significant (*p* ˃ 0.05).

A sensitivity analysis was performed by refitting the model after excluding non-significant explanatory variables (NRS for LBP and age). The final reduced regression model (Table 4) explained 47.7% of the variance in QoL (*p* < 0.001), with an adjusted R^2^ of 42.3%. The three baseline explanatory variables retained in the reduced model (thoracolumbar/lumbar Cobb angle, BMI, and DASS-Stress) remained significantly associated with QoL. Thoracolumbar/lumbar Cobb angle showed the strongest association with QoL (β = 0.448, *p* < 0.01), followed by DASS-Stress (β = −0.379, *p* < 0.05) and BMI (β = 0.362, *p* < 0.05). In this final model, a one-unit increase in thoracolumbar/lumbar Cobb angle and BMI were associated with an increase in QoL scores of 0.016 and 0.037 points, respectively, whereas a one-unit increase in DASS-Stress was associated with a 0.025-point decrease in QoL scores. No multicollinearity was detected among explanatory variables (VIF < 1.1).

## 4. Discussion

The objective of this study was to improve understanding of the impact of idiopathic scoliosis on the QoL of adolescents. To achieve this, relationships between psychological and physical factors and their influence on the health-related QoL of Saudi AIS patients were explored. Thoracolumbar/lumbar Cobb angle, BMI, and stress symptoms at baseline are reported to be significant explanatory variables of QoL 12 months after a baseline survey, explaining almost half the variance in its improvement. Accordingly, both physical and psychological domains must be considered when managing AIS. Such an approach is consistent with a biopsychosocial model for care which recognizes that biological, psychological, and social factors interact in influencing health outcomes and patient well-being [20]. However, given the modest sample size relative to the number of candidate predictors, the regression findings should be interpreted as exploratory and viewed with appropriate caution.

A notable finding of this study is the unexpected association between higher thoracolumbar/lumbar Cobb angles and improved QoL after 12 months. Although scoliosis severity has been typically linked to poorer outcomes, results are inconsistent [21,22,23]. While these factors were not directly assessed in the present study, previous research suggests several potential explanations for these findings. Some adolescents with moderate to severe spinal curvature may adapt psychologically over time, reporting stable or even improved QoL when well supported through clinical monitoring, physiotherapy, or bracing, as well as strong family and social support systems [24]. Brace and ongoing physiotherapy may reduce pain and provide a sense of reassurance—especially bracing, which has been associated with significantly lower spinal pain intensity and disability in the thoracic and lumbar regions [25]. Because 14.3% of our study participants reported using braces, their having done so may have contributed to improved QoL. Selection bias may also have contributed, as participants who completed follow-up assessments may have been more engaged in their care and better adjusted to their condition than those who lost to follow-up.

Sociocultural factors may have influenced the relationship between curve severity and QoL in this study’s cohort. While concerns related to cosmetic appearance and body image have been frequently reported [26], their relative importance varies across populations. Fear of surgery may further complicate self-reports, with some adolescents reporting higher QoL to minimize the perceived need for surgical intervention [27]. The absence of significant mental health problems (stress, anxiety, or depression) in this study cohort may have also contributed to more favorable QoL improvement [28]. Collectively, these discrepancies suggest that the relationship between curve severity and QoL is multifactorial. Further qualitative studies are needed to more fully investigate the roles of culture, psyche, and clinical influences on QoL.

BMI emerged as a strong explanatory variable of QoL in this sample, with adolescents with a lower BMI reporting lower QoL. Higher BMI has been associated with a lower prevalence of spinal deformities [29]. This finding is clinically relevant, because most scoliosis research has focused on obesity as a risk factor [30], leaving the impact of low BMI relatively underexplored [31]. Underweight adolescents may also face heightened body-image dissatisfaction and social stigma, which can increase the psychosocial burden of AIS [32]. Stress at baseline is also reported to be negatively associated with QoL in patients 12 months on from a baseline survey—a finding consistent with evidence suggesting that psychological distress affects outcomes in AIS [33]. Although DASS-Stress was not associated with QoL in the unadjusted analyses, it emerged as an important factor in the adjusted multivariable model. Given the exploratory nature of the analysis and the modest sample size, this finding should be interpreted cautiously and requires confirmation in larger prospective studies. Collectively, these findings improve understanding of the broader impacts of body weight on psychosocial well-being and QoL.

### 4.1. Clinical and Research Implications

Implications of these results for clinical practice include that: (1) AIS evaluation should extend beyond radiographic measures of curve severity. Routine screening for nutritional status (BMI) and psychological distress (particularly stress) should be integrated into clinical procedures. Early detection of stress symptoms allows for a more timely referral to psychological support services. (2), Underweight adolescents should be prioritized for dietary education and physical activity programs that promote strength and functional resilience. Results also identify the need for more research involving, and for example, intervention studies that evaluate whether psychosocial and nutritional interventions can meaningfully improve long-term QoL in AIS.

### 4.2. Strengths and Limitations

A study strength is its prospective cohort design, which enabled an exploration of temporal associations between measures at baseline and those at 12 months. The use of validated Arabic-language instruments ensured methodological rigor and cultural relevance, and the high follow-up rate (71%) adds confidence to reported findings. The integration of psychological and physical explanatory variables in questionnaires also provides a more holistic understanding of QoL determinants in AIS. These strengths aside, some study limitations are acknowledged. No separate sample size calculation was performed for the longitudinal phase. Further, the regression analyses were based on only 33 participants with complete follow-up data. Consequently, the modest follow-up sample may have limited statistical power and increased the risk of overfitting, affecting the stability of the findings. Baseline QoL scores were not included as covariates due to sample size constraints; therefore, the observed associations should be interpreted cautiously. Additionally, although no significant baseline differences were observed between responders and non-responders, attrition bias cannot be entirely excluded. The use of self-reported measures also may have introduced reporting bias, though independent completion of questionnaires was encouraged. Although DASS-21 has demonstrated acceptable psychometric properties in adolescent populations, its validity in younger participants at the lower end of the age range included in this study may be less well established. Generalizability also may be limited to Saudi adolescents receiving care in tertiary centers; these findings may differ in community or international populations. Finally, unmeasured factors such as socioeconomic status, brace compliance, or parental support may have influenced QoL trajectories.

## 5. Conclusions

We found that baseline thoracolumbar/lumbar Cobb angle, BMI, and stress symptoms were associated with QoL after 12 months in Saudi adolescents with AIS. These findings highlight the importance of addressing both physical and psychosocial factors in AIS management, in which structural deformity and psychological well-being are addressed in unison. By adopting a multidimensional and culturally sensitive approach, clinicians can improve long-term QoL and overall outcomes for adolescents living with scoliosis.

## Figures and Tables

**Table 1:** Participant baseline characteristics (n = 69).

Variable ^a^	Respondents (n = 49)	Non Respondents (n = 20)	*p*
Age, years	15 (3)	16 (4)	0.274
Female gender, n (%)	41 (83.7)	18 (90)	0.712
Smoking status, n (%)			0.290
Non-smoker	49 (100)	19 (95)	
Smoker	0 (0)	1 (5)	
Comorbidities, n (%)			
Diabetes	1 (2)	0 (0)	0.710
Cardiovascular conditions	0 (0)	0 (0)	1.000
Respiratory conditions	1 (2)	0 (0)	0.710
NRS LBP intensity (0–10)	5 (5)	5 (4)	0.621
Analgesics usage, n (%)	7 (14.3)	5 (25)	0.287
Time since diagnosis, years	1 (2.3)	0.88 (2.7)	0.521
Use of a Thoracolumbosacral orthosis (TLSO), n (%)	7 (14.3)	2 (10)	0.632
Having PT sessions, n (%)			
Type of PT			0.272
General exercise	9 (18.4)	2 (10)	
Home exercise (stretching)	0 (0)	1 (5)	
Electric KKT	1 (2)	0 (0)	
Schroth method exercise	1 (2)	1 (5)	
Other	0 (0)	1 (5)	
None	38 (77.6)	15 (75)	
Physical outcomes			
Beighton score (0–9)	6 (4)	5.5 (5)	0.698
Height (cm)	156 (12.5)	160 (11.5)	0.508
Weight (kg)	43 (14.5)	47.5 (14.5)	0.175
BMI (kg m^−2^)	17.7 (4.9)	19.5 (5.8)	0.473
Underweight, n (%)	15 (30.6)	3 (15)	
Healthy weight, n (%)	24 (49.0)	14 (70)	
Overweight, n (%)	4 (8.2)	1 (5)	
Obesity, n (%)	4 (8.2)	2 (10)	
Severe obesity, n (%)	2 (4)	0 (0)	
Participants involved in any weekly PA, n (%)	8 (16.3)	4 (20)	0.734
IPAQ	330 (1074)	346.5 (703)	0.979
Physical activity classification, IPAQ, n (%)			0.304
Low	33 (67.3)	14 (70)	
Moderate	11 (22.4)	6 (30)	
High	5 (10.2)	0 (0)	
Psychological outcomes			
DASS-21 total (0–63)	34 (28)	27(30)	0.206
DASS-Anxiety (0–21)	10 (13)	4 (8)	0.116
DASS-Depression (0–21)	4 (16)	4 (15)	0.538
DASS-Stress (0–21)	16 (10)	13 (18)	0.306

Median (interquartile range) or frequency (%). **^a^** Independent Samples *t*-tests or Mann–Whitney *U* tests for continuous variables, χ^2^-test or Fisher’s Exact Tests for categorical variables (as appropriate). BMI: Body mass index; DASS: Depression Anxiety Stress Scales; IPAQ: International Physical Activity Questionnaire; KKT: Khan kinetic treatment; LBP: low back pain; NRS: Numeric Rating Scale; PA: physical activity; PT: physical therapy.

**Table 2:** Bivariate linear regression analyses of associations between baseline sociodemographic, clinical, physical, and psychological variables and 12-month quality of life (SRS-22).

Independent Variable	n	R^2^ (%)	F	Standardized *β*	*p*
Sociodemographic variable					
Time since diagnosis	49	0.1	0.1	−0.037	0.804
Age	49	0.8	0.4	0.008	0.549
BMI	49	9.5	4.9	0.308	0.031 *
Clinical and physical variables					
NRS LBP	49	7.4	3.7	−0.273	0.058
Beighton score	49	0.3	0.2	0.056	0.702
Cobb angle, thoracic	28	2.0	0.5	−0.140	0.477
Cobb angle, thoracolumbar/lumbar	33	20.1	7.8	0.448	0.009 **
IPAQ	49	1.6	0.8	0.126	0.387
Psychological variable					
DASS-Total	49	1.5	0.7	−0.121	0.406
DASS-Anxiety	49	1.4	0.7	−0.119	0.416
DASS-Depression	49	0.0	0.02	−0.018	0.902
DASS-Stress	49	2.9	1.4	−0.171	0.239

* *p* < 0.05; ** *p* < 0.01. BMI: Body mass index; DASS: Depression Anxiety Stress Scales; IPAQ: International Physical Activity Questionnaire; LBP: low back pain; n: sample size; NRS: Numeric Rating Scale; SRS-22: Scoliosis Research Society-22 questionnaire.

**Table 3:** Results of multiple linear regression for the association between quality of life in adolescents with idiopathic scoliosis after 12 months (n = 33).

Model Term	Coefficients (Std Error)	95% CI	Standardized *β*	R^2^ (%)	F	*p*
Model ^a^				53.3	6.2	˂0.001 **
Intercept	3.213 (0.570)	2.042 to 4.383				˂0.001 **
Independent variables						
Cobb angle, thoracolumbar/lumbar	0.017 (0.005)	0.007 to 0.027	0.476			0.002 **
BMI	0.035 (0.015)	0.004 to 0.065	0.342			0.026 *
DASS-Stress	−0.021 (0.010)	−0.040 to −0.001	−0.309			0.041 *
NRS LBP	−0.047 (0.030)	−0.109 to 0.015	−0.235			0.132
Age	−0.008 (0.044)	−0.097 to 0.082	−0.028			0.865

^a^ Multiple linear regression analysis; n = 33. Thoracolumbar/lumbar Cobb angle data at follow-up were available for 33 of the 49 participants and analyses were therefore based on this subset. Dependent variable: AIS-related Quality of life (SRS-22) at 12 months. Variables entered into this analysis included pain intensity, physical activity, age, gender, BMI, time since diagnosis, stress, anxiety, depression, thoracolumbar/lumbar Cobb angle, thoracic Cobb angle, and joint hypermobility. No collinearity was found between variables in this model (VIF ˂ 1.5). Accuracy: Adjusted R^2^ (%) = 44.6%. Statistically significant at * *p* < 0.05, and ** *p* < 0.01. BMI: Body mass index; CI: Confidence Interval; DASS: Depression Anxiety Stress Scales; LBP: low back pain; NRS: Numeric Rating Scale.

**Table 4:** Sensitivity analysis: reduced multiple linear regression model of baseline factors associated with 12-month quality of life (SRS-22) in adolescents with idiopathic scoliosis (n = 33).

Model Term	Coefficients (Std Error)	95% CI	Standardized *β*	R^2^ (%)	F	*p*
Model ^a^				47.7	8.8	˂0.001 **
Intercept	2.996 (0.336)	2.309 to 3.683				˂0.001 **
Independent variables						
Cobb angle, thoracolumbar/lumbar	0.016 (0.005)	0.006 to 0.026	0.448			0.003 **
BMI	0.037 (0.014)	0.008 to 0.065	0.362			0.013 *
DASS-Stress	−0.025 (0.009)	−0.043 to −0.006	−0.379			0.011 *

^a^ Multiple linear regression analysis; n = 33. Thoracolumbar/lumbar Cobb angle data at follow-up were available for 33 of the 49 participants and analyses were therefore based on this subset. Dependent variable: AIS-related Quality of life (SRS-22) at 12 months. Variables entered into this analysis were BMI, stress, and thoracolumbar/lumbar Cobb angle. No collinearity was found between variables in this model (VIF ˂ 1.1). Accuracy: adjusted R^2^ (%) = 42.3%. Statistically significant at * *p* < 0.05, and ** *p* < 0.01. BMI: Body mass index; CI: Confidence Interval; DASS: Depression Anxiety Stress Scales.

## Data Availability

The datasets generated during this study are available from the corresponding author on reasonable request.

## Appendix A

**Table A1:** Correlations between sociodemographic, clinical, physical, and psychosocial outcomes at baseline and quality of life after 12 months (SRS-22) in adolescents with idiopathic scoliosis.

Variable	AIS (n)	Correlation with SRS-22	
		(*r*) ^a^	*p*
Sociodemographic			
Time since diagnosis	49	0.037	0.804
Gender	49	−0.043 ^†^	0.767
Age	49	0.088	0.549
BMI	49	0.228	0.115
Clinical and physical			
NRS LBP	49	−0.228	0.114
Beighton score	49	0.098	0.503
Cobb angle, thoracic	28	−0.054	0.783
Cobb angle, thoracolumbar/lumbar	33	0.361	0.039 *
IPAQ	49	0.177	0.222
Psychological			
DASS-total	49	−0.054	0.713
DASS-Anxiety	49	−0.116	0.428
DASS-Depression	49	−0.020	0.891
DASS-Stress	49	−0.098	0.502

^a^ *r* Spearman’s rho, for non-normally distributed variables, unless otherwise stated. ^†^ *r* Point Biserial Correlation. ***** *p* < 0.05. AIS: Adolescents with idiopathic scoliosis; BMI: body mass index; DASS: Depression Anxiety Stress Scales; IPAQ: International Physical Activity Questionnaire; LBP: low back pain; n: sample size; NRS: Numeric Rating Scale; SRS-22: Scoliosis Research Society-22 questionnaire.

## References

[1] Cheng J.C., Castelein R.M., Chu W.C., Danielsson A.J., Dobbs M.B., Grivas T.B., Gurnett C.A., Luk K.D., Moreau A., Newton P.O. (2015). Adolescent idiopathic scoliosis. Nat. Rev. Dis. Prim..

[2] AlAssiri S.S., Aleissa S.I., Alhandi A.A., Konbaz F.M., Alhelal F., Abaalkhail M., Al-Annaim M.M., Alhabeeb A., Alshehri K.M. (2022). Prevalence and Predictors of Scoliosis and Back Pain in 591 Adolescents: A Randomized, Stratified, Cross-Sectional Study in Riyadh, Saudi Arabia. Cureus.

[3] Corrêa L.A., Mathieson S., Meziat-Filho N.A., Reis F.J., Ferreira A.S., Nogueira L.A. (2022). Which psychosocial factors are related to severe pain and functional limitation in patients with low back pain?: Psychosocial factors related to severe low back pain. Braz. J. Phys. Ther..

[4] Dunn M., Rushton A.B., Mistry J., Soundy A., Heneghan N.R. (2024). The biopsychosocial factors associated with development of chronic musculoskeletal pain. An umbrella review and meta-analysis of observational systematic reviews. PLoS ONE.

[5] Mitsiaki I., Thirios A., Panagouli E., Bacopoulou F., Pasparakis D., Psaltopoulou T., Sergentanis T.N., Tsitsika A. (2022). Adolescent idiopathic scoliosis and mental health disorders: A narrative review of the literature. Children.

[6] Pezham H., Babaee T., Bagheripour B., Asgari M., Jiryaei Z., Vahab Kashani R., Rahgozar M., Arazpour M. (2022). Stress level and quality of life of adolescents with idiopathic scoliosis during brace treatment. Turk. J. Phys. Med. Rehabil..

[7] Ituen O.A., Akwaowo C.D., Ferguson G., Duysens J., Smits-Engelsman B. (2025). Impact of generalized joint hypermobility on quality of life and physical activity in school-aged children: A longitudinal study. BMC Musculoskelet. Disord..

[8] Jada A., Mackel C.E., Hwang S.W., Samdani A.F., Stephen J.H., Bennett J.T., Baaj A.A. (2017). Evaluation and management of adolescent idiopathic scoliosis: A review. Neurosurg. Focus..

[9] von Elm E., Altman D.G., Egger M., Pocock S.J., Gøtzsche P.C., Vandenbroucke J.P. (2007). Strengthening the Reporting of Observational Studies in Epidemiology (STROBE) Statement: Guidelines for Reporting Observational Studies. BMJ.

[10] Boonstra A.M., Preuper H.R.S., Balk G.A., Stewart R.E. (2014). Cut-off points for mild, moderate, and severe pain on the visual analogue scale for pain in patients with chronic musculoskeletal pain. Pain.

[11] Castori M., Tinkle B., Levy H., Grahame R., Malfait F., Hakim A. (2017). A framework for the classification of joint hypermobility and related conditions. Am. J. Med. Genet. Part C Semin. Med. Genet..

[12] Tofts L.J., Simmonds J., Schwartz S.B., Richheimer R.M., O’Connor C., Elias E., Engelbert R., Cleary K., Tinkle B.T., Kline A.D. (2023). Pediatric joint hypermobility: A diagnostic framework and narrative review. Orphanet J. Rare Dis..

[13] Centers for Disease Control and Prevention Child and Teen BMI Categories. https://www.cdc.gov/bmi/child-teen-calculator/bmi-categories.html.

[14] Al-Hazzaa H.M. (2007). Health-enhancing physical activity among Saudi adults using the International Physical Activity Questionnaire (IPAQ). Public Health Nutr..

[15] Negrini S., Donzelli S., Aulisa A.G., Czaprowski D., Schreiber S., de Mauroy J.C., Diers H., Grivas T.B., Knott P., Kotwicki T. (2018). 2016 SOSORT guidelines: Orthopaedic and rehabilitation treatment of idiopathic scoliosis during growth. Scoliosis Spinal Disord..

[16] Taouk M., Lovibond P., Laube R. (2001). Psychometric properties of an Arabic version of the Depression Anxiety Stress Scales (DASS21). Report for New South Wales Transcultural Mental Health Centre, Cumberland Hospital, Sydney.

[17] Haidar R.K., Kassak K., Masrouha K., Ibrahim K., Mhaidli H. (2015). Reliability and validity of an adapted Arabic version of the Scoliosis Research Society-22r questionnaire. Spine J..

[18] Alhafdhi N., Alimam D., Alrashed Alhumaid L., Alamrani S., Bin Shebreen A. (2026). Multidimensional clinical profiles of Saudi adolescents with idiopathic scoliosis: An exploratory cross-sectional study. Ann. Med..

[19] Field A. (2009). Discovering Statistics Using SPSS.

[20] Bolton D. (2023). A revitalized biopsychosocial model: Core theory, research paradigms, and clinical implications. Psychol. Med..

[21] Brandwijk A.C., Heemskerk J.L., Willigenburg N.W., Altena M.C., Kempen D.H.R. (2023). Health-related quality of life of adult, non-surgically treated patients with idiopathic scoliosis and curves above 45°: A cross-sectional study at an average follow-up of 30 years. Eur. Spine J..

[22] Erwin J., Carlson B.B., Bunch J., Jackson R.S., Burton D. (2020). Impact of unoperated adolescent idiopathic scoliosis in adulthood: A 10-year analysis. Spine Deform..

[23] Torén S., Diarbakerli E. (2022). Health-related quality of life in adolescents with idiopathic scoliosis: A cross-sectional study including healthy controls. Eur. Spine J..

[24] Glahn Castille M., Resendiz Ortega S. (2025). Strong support systems foster positive self-image in patients with scoliosis. Discov. Ment. Health.

[25] Théroux J., Le May S., Hebert J.J., Labelle H. (2017). Back pain prevalence is associated with curve-type and severity in adolescents with idiopathic scoliosis: A cross-sectional study. Spine J..

[26] Carrasco M.I., Ruiz M.C. (2014). Perceived self-image in adolescent idiopathic scoliosis: An integrative review of the literature (Imagen percibida en la escoliosis idiopática adolesscente: Revision integrative de la literatura). Rev. Esc. Enferm. USP.

[27] Motyer G.S., Kiely P.J., Fitzgerald A. (2022). Adolescents’ experiences of idiopathic scoliosis in the presurgical period: A Qualitative Study. J. Pediatr. Psychol..

[28] Leszczewska J., Czaprowski D., Pawłowska P., Kolwicz A., Kotwicki T. (2012). Evaluation of the stress level of children with idiopathic scoliosis in relation to the method of treatment and parameters of the deformity. Sci. World J..

[29] Hershkovich O., Friedlander A., Gordon B., Arzi H., Derazne E., Tzur D., Shamiss A., Afek A. (2014). Association between body mass index, body height, and the prevalence of spinal deformities. Spine J..

[30] Catanzariti J.F., Rimetz A., Genevieve F., Renaud G., Mounet N. (2023). Idiopathic adolescent scoliosis and obesity: Prevalence study. Eur. Spine J..

[31] Scaturro D., Balbo A., Vitagliani F., Stramazzo L., Camarda L., Letizia Mauro G. (2022). Is there a relationship between idiopathic scoliosis and body mass? A Scoping Review. Nutrients.

[32] Moehlecke M., Blume C.A., Cureau F.V., Kieling C., Schaan B.D. (2020). Self-perceived body image, dissatisfaction with body weight and nutritional status of Brazilian adolescents: A nationwide study. J. Pediatr..

[33] Hong K.S., Nguyen T.B.G., Trinh Q.D., Nguyen H.N., Pham V.M. (2025). Stress level of adolescent with idiopathic scoliosis using braces for the treatment: A descriptive study in Vietnam. JPO J. Prosthet. Orthot..

